# Positive selection on panpulmonate mitogenomes provide new clues on adaptations to terrestrial life

**DOI:** 10.1186/s12862-016-0735-8

**Published:** 2016-08-22

**Authors:** Pedro E. Romero, Alexander M. Weigand, Markus Pfenninger

**Affiliations:** 1 Senckenberg Biodiversity and Climate Research Centre (BiK-F), Senckenberganlage 25, 60325 Frankfurt am Main, Germany; 2 Institute for Ecology, Evolution & Diversity, Faculty of Biological Sciences, Goethe University Frankfurt, Max-von-Laue-Straße 13, 60438 Frankfurt am Main, Germany; 3 Museo de Historia Natural, Universidad Nacional Mayor de San Marcos, Av. Arenales 1256, Apartado 14-0434, Lima 14, Peru; 4 Aquatic Ecosystem Research, University of Duisburg-Essen, Universitätsstraße 5, 45141 Essen, Germany; 5 Centre for Water and Environmental Research (ZWU), University of Duisburg-Essen, Universitätsstraße 2, 45117 Essen, Germany

**Keywords:** Codon models, Land invasion, Mitogenomics, Panpulmonata, Positive selection

## Abstract

**Background:**

Transitions from marine to intertidal and terrestrial habitats resulted in a significant adaptive radiation within the Panpulmonata (Gastropoda: Heterobranchia). This clade comprises several groups that invaded the land realm independently and in different time periods, e.g., Ellobioidea, Systellomatophora, and Stylommatophora. Thus, mitochondrial genomes of panpulmonate gastropods are promising to screen for adaptive molecular signatures related to land invasions.

**Results:**

We obtained three complete mitochondrial genomes of terrestrial panpulmonates, i.e., the ellobiid *Carychium tridentatum*, and the stylommatophorans *Arion rufus* and *Helicella itala*. Our dataset consisted of 50 mitogenomes comprising almost all major panpulmonate lineages. The phylogenetic tree based on mitochondrial genes supports the monophyly of the clade Panpulmonata. Terrestrial lineages were sampled from Ellobioidea (1 sp.) and Stylommatophora (9 spp.). The branch-site test of positive selection detected significant non-synonymous changes in the terrestrial branches leading to *Carychium* (Ellobiodea) and Stylommatophora. These convergent changes occurred in the *cob* and *nad5* genes (OXPHOS complex III and I, respectively).

**Conclusions:**

The convergence of the non-synonymous changes in *cob* and *nad5* suggest possible ancient episodes of positive selection related to adaptations to non-marine habitats. The positively selected sites in our data are in agreement with previous results in vertebrates suggesting a general pattern of adaptation to the new metabolic requirements. The demand for energy due to the colonization of land (for example, to move and sustain the body mass in the new habitat) and the necessity to tolerate new conditions of abiotic stress may have changed the physiological constraints in the early terrestrial panpulmonates and triggered adaptations at the mitochondrial level.

**Electronic supplementary material:**

The online version of this article (doi:10.1186/s12862-016-0735-8) contains supplementary material, which is available to authorized users.

## Background

The transition from water to land is a fascinating evolutionary issue. The realm change occurred several times and across different taxa, from microorganisms to lichens and green plants, and later, arthropods, mollusks, annelids and vertebrates [1]. The multiple transitions required modifications on several systems and organs previously adapted to aquatic habitats. For example, the presence of internal gas exchangers (lungs) to uptake oxygen, or different skin modifications as cuticle and keratin layers to decrease evaporation rates. Other examples include the production of novel compounds (e.g., uric acid and urea) to excrete nitrogen, and the presence of a skeleton and thick body muscles to support the body mass [2].

The vast majority of mollusks are marine, but several lineages within the clade Gastropoda have conquered freshwater and terrestrial habitats [3], e.g., Neritimorpha, Cyclophoroidea, Littorinoidea, Rissooidea, and Panpulmonata [4]. Most terrestrial mollusks belong to the clade Panpulmonata (Gastropoda: Euthyneura) [5]. The invasion of the non-marine realms in Panpulmonata occurred multiple times independently in the clades Ellobioidea, Hygrophila, Stylommatophora and Systellommatophora [6–8]. For this reason, panpulmonates are a suitable model to study parallel adaptations to new environments, in particular to land.

Examples from invertebrates like the Collembola (Arthropoda: Hexapoda) showed that adaptive changes during terrestrialization have probably occurred in genes related to ion transport, homeostasis, immune response and development [9]. On the other hand, several examples from vertebrates showed various molecular mechanisms of adaptation: duplication and functional diversification of keratin genes [10], expansion of genes encoding olfactory receptors to detect airborne ligands [11], and positive selection on either nuclear genes involved in the urea cycle [12], or mitochondrial genes responding to the increase of oxygen during the Devonian [13]. However, the genomic basis of the transition to the land in panpulmonates is still unknown.

The animal mitochondrial genome encodes 13 proteins that are involved in the production of almost all energy in the eukaryotic cells. These proteins belong to four of the five complexes of the oxidative phosphorylation (OXPHOS) pathway and are under high functional constraints [14]. For example, inefficiencies caused by amino acid changes in these proteins alter the OXPHOS performance, and produce reactive oxygen species (ROS), i.e., molecules that lead to cellular damage and metabolism disruption [15]. In addition, amino acid substitutions have been shown to improve aerobic capacity and adaptation to new environments [16].

Several studies have reported non-neutral changes in each of the 13 mitochondrial genes [17]. Cytochrome c oxidase genes *cox1* and *cox3* from the freshwater fish *Poecilia* spp. present substitutions involved in the adaptation to toxic (H_2_S-rich) environments. These substitutions trigger conformational changes that block the uptake of H_2_S [18]. In addition, repeated selection at the same structural amino acid location has been found in the mitochondrial complex I from other fish, rodents and snakes [19]. The changes likely impacted the biomechanical apparatus that affect the electrochemical gradient in the mitochondria [17]. Moreover, cytochrome b (*cob*) in whales demonstrates several signatures of positive selection, in comparison to other artiodactyls. The adaptive changes in *cob* have been related to changes in the demand of metabolic processes during cetacean cladogenesis and the transition from land to water habitats [20].

Mitochondrial genomes of euthyneuran gastropods represent a promising dataset to screen for adaptive signatures related to water-to-land transitions. As mentioned above, this clade contains terrestrial and intertidal panpulmonates (e.g., Stylommatophora, Systellommatophora, and Ellobiodea), and also freshwater taxa (Hygrophila) as well as other marine clades (e.g., Euopisthobranchia, Nudipleura). Here, we sequenced and annotated three new panpulmonate mitogenomes from the terrestrial ellobid *Carychium tridentatum* (Risso, 1826) and the stylommatophorans *Arion rufus* (Linnaeus, 1758) and *Helicella itala* (Linnaeus, 1758). We used these new mitogenomes in addition to 47 already published euthyneuran mitogenomes, to reconstruct the phylogenetic relationships of Euthyneura. Finally, we evaluate the magnitude of selective pressures that occurred on the branches leading to terrestrial taxa.

## Results and discussion

### Characteristics of the new panpulmonate mitogenomes

The length of the three new mitochondrial genomes from the terrestrial panpulmonates *Carychium tridentatum* (Ellobiidae, Ellobioidea), *Arion rufus* and *Helicella itala* (Arionidae and Hygromiidae, Stylommatophora) are 13908 bp, 14321 bp, and 13966 bp, respectively. The three mitogenomes all encode for 13 protein-coding genes (PCG), 22 tRNAs, and 2 rRNAs, as reported for most other animal mitogenomes [21]. A detailed overview of the gene annotations can be found in Table 1. Nine genes are encoded on the major strand: *cox1*, *cox2*, *cob*, *nad1*, *nad2*, *nad4*, *nad4L*, *nad5* and *nad6*; while four are encoded in the minor strand: *atp6*, *atp8*, *cox3* and *nad3*. The gene arrangement is similar to other panpulmonates [21, 22]. Basically, the coding-genes are organized as follows: *cox1*–*nad6*–*nad5*–*nad1*–*nad4L*–*cob*–*cox2*–*atp8*–*atp6*–*nad3*–*nad4*–*cox3*–*nad2*. The cluster *cox2*-*atp8*-*atp6* is conserved among other gastropods and cephalopods [20]. However, in *A. rufus*, we found the small rRNA subunit between *cox2* and *atp8* (*cox2*-*rrnS*–*atp8*–*atp6*). Furthermore, clusters *trnD*-*trnC*-*trnF* and *trnY*-*trnW*-*trnG*-*trnH*-*trnQ*-*trnL2* are typical in Ellobioidea and Systellomatophora [22]. We found these clusters in the ellobiid *C. tridentatum*, but also in the stylommatophorans *H. itala*, and *A. rufus*, the latter with a slight modification (*trnW*-*trnY*).

**Table 1:** Gene features in the three new panpulmonate mitogenomes

		Ellobioidea				Stylommatophora							
	Coding	*Carychium tridentatum*				*Arion rufus*				*Helicella itala*			
Gene	strand			Start	Stop			Start	Stop			Start	Stop
		From	To	codon	codon	From	To	codon	codon	From	To	codon	codon
*cox1*	+	1	1536	TTG	TAA	1	1530	TTG	TAA	1	1530	ATG	TAA
*trnV*	+	1529	1591	-	-	1538	1600	-	-	1527	1587	-	-
*rrnL*	+	1679	2645	-		1690	2578	-	-	1699	2583	-	-
*trnL1*	+	2588	2653	-	-	2592	2654	-	-	2595	2655	-	-
*trnP*	+	**2712**	**2773**	-	-	2657	2721	-	-	2656	2712	-	-
*trnA*	+	**2650**	**2712**	-	-	2739	2803	-	-	2714	2775	-	-
*nad6*	+	2778	3233	ATT	TAA	2838	3278	ATA	TAG	2776	3249	TTG	TAA
*nad5*	+	3258	4889	ATT	TAG	3238	4920	ATA	TAA	3239	4915	TTG	TAA
*nad1*	+	4867	5775	TTG	TAA	4911	5813	ATG	TAG	4867	5796	ATT	TAA
*nad4l*	+	5776	6114	GTG	TAA	5822	6124	ATA	TAG	5787	6098	ATT	TAG
*cob*	+	6056	7177	ATT	TAG	6103	7182	TTG	TAA	6113	7187	TTG	T
*trnD*	+	7175	7224	-	-	7184	7248	-	-	7188	7242	-	-
*trnC*	+	7228	7290	-	-	7284	7342	-	-	7243	7303	-	-
*trnF*	+	7291	7354	-	-	7346	7409	-	-	7307	7368	-	-
*cox2*	+	7340	8053	TTG	TAA	7412	8080	ATG	TAG	7369	8067	GTG	TAG
*trnY*	+	8028	8079	-	-	**8207**	**8270**	-	-	8042	8101	-	-
*trnW*	+	8080	8140	-	-	**8082**	**8149**	-	-	8097	8157	-	-
*trnG*	+	8150	8197	-	-	8465	8527	-	-	8158	8218	-	-
*trnH*	+	8199	8260	-	-	8522	8591	-	-	8215	8274	-	-
*trnQ*	-	8292	8354	-	-	**8672**	**8736**	-	-	8275	8332	-	-
*trnL2*	-	8355	8419	-	-	**9557**	**9624**	-	-	8333	8388	-	-
*atp8*	-	8394	8570	TTG	TAG	**9578**	**9775**	ATG	TAG	8360	8614	ATG	TAA
*trnN*	-	8571	8637	-	-	**9781**	**9843**	-	-	8615	8677	-	-
*atp6*	-	8638	9282	ATG	TAA	**9836**	**10489**	TTG	TAA	8679	9332	ATA	TAA
*trnR*	-	9283	9343	-	-	**10490**	**10555**	-	-	9330	9389	-	-
*trnE*	-	9344	9408	-	-	**8589**	**8655**	-	-	9390	9449	-	-
*rrnS*	-	9408	10086	-	-	**8822**	**9477**	-	-	9449	10126	-	-
*trnM*	+	10108	10176	-	-	**9491**	**9553**	-	-	10145	10210	-	-
*nad3*	-	10172	10528	ATA	TAA	10557	10904	ATA	TAA	10188	10556	ATG	TAA
*trnS2*	-	10540	10595	-	-	10914	10979	-	-	10554	10605	-	-
*trnS1*	+	10598	10653	-	-	10993	11053	-	-	10683	10734	-	-
*nad4*	+	10653	11960	TTG	TAA	11041	12444	ATT	TAA	10723	12036	ATC	TAG
*trnT*	-	11983	12047	-	-	12391	12455	-	-	12040	12101	-	-
*cox3*	-	12038	12826	ATG	TAG	12436	13233	ATT	TAA	12082	12882	ATA	TAA
*trnI*	+	12871	12933	-	-	13280	13342	-	-	12921	12980	-	-
*nad2*	+	12933	13871	ATT	TAG	13307	14230	TTG	TAA	12969	13913	ATT	TAG
*trnK*	+	13860	2	-	-	14262	8	-	-	13914	7	-	-

Annotations were performed in the MITOS server using default parameters, and then manually refined in Geneious R7. +/− signs indicate the sense of each annotation. Gene rearrangements with respect to the other two mitogenomes are indicated in bold

The total length of the PCG is 10923 bp in *C. tridentatum*, 10935 bp in *A. rufus*, and 11071 bp *H. itala*. The GC-content of the PCG is approximately 30 %, being slightly higher in *H. itala* (34 %). PCG start with five different initiation codons: ATA, ATG, ATT, GTG, TTG. Finally, an AT-rich intergenic spacer between *cox3* and *trnI* has been proposed as the potential origin of replication (POR) in other euthyneurans [22, 23]. We found the same intergenic region in each of the three new mitogenomes. The AT-mean value in the potential POR region was 83 %.

### Phylogenetic analyses

**Fig. 1:**
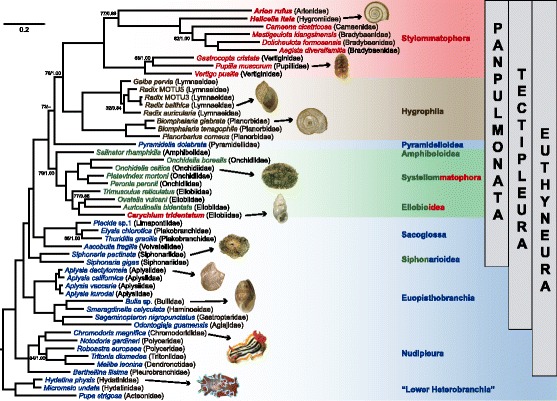
Phylogenetic tree of the Euthyneura based on mitochondrial genomes. Support for all branches is higher than 95/0.99 (bootstrap/posterior probability) unless otherwise indicated. Habitats are represented by colors following the scheme proposed by Schrödl [30]: marine (*blue*), intertidal (*green*), freshwater (*brown*), and terrestrial (*red*). Mixed colors indicate clades that occur in multiple habitats. Terrestrial taxa (*Arion*, *Carychium* and *Helicella*) with new mitogenomic data are highlighted in bold letters. Photo credits: Natural Museum Rotterdam (*A. dactylomela*: NMR40266, *B. glabrata* NMR81004, *Bulla* sp. NMR52020, *C. tridentatum* NMR80709, *H. itala*: NMR59501, *O. celtica* NMR69303, *P. muscorum*: NMR48018, *R. balthica*: NMR76454, *S. pectinata* NMR78115), Stephen Childs (*C. magnifica*)/CC BY 2.0, Samuel Chow (*H. physis*)/CC BY 2.0

Our reconstructed tree is congruent with previous comprehensive phylogenetic analyses in Euthyneura, using a combination of mitochondrial and nuclear genes [5], and phylogenomics [3, 24] (Fig. 1). Tectipleura (Euopisthobranchia + Panpulmonata) [25] are highly supported in both ML and BI analyses. The clade Nudipleura is monophyletic and within this clade, Pleurobranchidae (*Berthellina*) is the sister group of Nudibranchia, as proposed by Göbbeler et al. [26]. In addition, there is high support for the clade Euopisthobranchia. This clade was defined by Jörger et al. [5] reuniting the clades Umbraculoidea, Anaspidea, Runcinacea, Pteropoda and Cephalaspidea. In our topology, Anaspidea (*Aplysia* spp.) and the cephalaspideans *Bulla*, *Odontoglaja*, *Sagaminopteron* and *Smaragdinella* conform a monophyletic group.

Previous mitochondrial phylogenetic reconstructions recovered the monophyly of the former accepted clade “Opisthobranchia” and the paraphyly of “Pulmonata” [22, 23, 27]. However, the topologies derived from mitogenomics have received criticism, for long-branch attraction (LBA) artifacts affecting the topologies in Heterobranchia. In these cases, long-branched stylommatophorans were recovered closer to the root of the clade while they appeared as derived in the nuclear topologies [28]. On the other hand, recent genomic evidence rejected “Opisthobranchia” in favor of Euopisthobranchia as the sister group of Panpulmonata. Phylogenetic reconstructions based on concatenated nuclear and mitochondrial genes [5, 7, 29] as well new phylogenomic studies [3, 24] recovered the paraphyly of “Opisthobranchia”, and support for Panpulmonata.

We were aware of these rooting issues; thus, we choose members of the “Lower Heterobranchia” as outgroup taxa. Our topology recovered monophyletic Panpulmonata and Euopisthobranchia as its sister group. The clade Panpulmonata, defined by Jörger et al. [5], comprises the clades Amphiboloidea, Ellobioidea, Glacidorboidea, Hygrophila, Siphonarioidea, Stylommatophora, and Systellomatophora plus Acochlidia and Sacoglossa, previously regarded as opisthobranchs [30]. Therefore, Panpulmonata possesses an extraordinary diversity in morphology (snails, slugs and intermediate forms), and habitats (marine, intertidal, freshwater and terrestrial).

Recently, the monophyly of “Pulmonata” has been challenged [30], i.e., evidence from phylogenomics did not recover “Pulmonata” as a monophyletic group [3]. In our tree, members from Amphibolidae (*Salinator*) and Pyramidelloidea (*Pyramidella*) appear between traditional “Pulmonata” clades, favoring Panpulmonata over “Pulmonata”. Our topology supports the Amphipulmonata clade (Ellobioidea + Systellomatophora) [29], and rejects the Geophila hypothesis (Stylommatophora + Systellomatophora) [30]. Finally, the association between Stylommatophora and Hygrophila has been also found using phylogenomic analyses [24], although with a small subset of euthyneuran taxa.

### Patterns of evolutionary rates

The relative evolutionary rates (RER) for amino acids were not equally distributed over the alignment (Additional file 1). The mean RER value in the *nad* genes was 2–3 times higher than in the *cox* genes. The RER for the *cox1* gene were below the mean rates in our dataset, indicating a higher number of conserved sites.

Stylommatophora (y = 0.6514x; *R*^2^ = 0.9641) along with Hygrophila (y = 0.6708x; *R*^2^ = 0.9770) presented the highest divergence slopes in our data (Additional file 2). This means that fewer nucleotide changes produced more amino acid changes in comparison to the other clades, i. e. non-synonymous changes are more frequent in both Stylommatophora and Hygrophila. Furthermore, Stylommatophora presented the highest absolute values for both nucleotide and amino acid divergence. This result explains the presence of long branches in this clade (Fig. 1).

Several hypotheses have been proposed to explain the extreme divergence found in the mitochondrial DNA of land snails (Stylommatophora) [31]: (1) exceptionally accelerated rate of evolution, (2) haplotype groups previously differentiated in isolated refuges getting into secondary contact, (3) natural (positive) selection preserving the variation, and (4) the particular population structure in pulmonates that allowed them to preserve ancient haplotypes. Accelerated evolution of the mtDNA has been found in several species of land snails and slugs (hypothesis 1). The evolutionary rate in the mtDNA (rRNA) of the snails *Euhadra* and *Mandarina* was 10 % per Ma [32, 33], and 5.2 % in the slug *Arion* [34]. However, this is not a general pattern for all pulmonates, for example the evolutionary rate of *Albinaria* and *Partula* was estimated to be 1–1.2 and 2.8 % per Ma, respectively [35]. The secondary contact after allopatric divergence of haplotypes (hypothesis 2) has been found in *Candidula* [36] and *Cepaea* [37]. Moreover, introgression of mitochondrial lineages as a result of hybridization has been observed in two species of the land snail genus *Trochulus* [38]. The effect of natural selection (hypothesis 3) shaping the genetic diversity has been proposed before for land snails [39] although it was considered to be uncommon [40]. However, a recent study has shown that mitochondrial DNA undergoes substantial amounts of adaptive evolution, especially in mollusks [41]. The particular demographic pattern of land snails that produces highly structured populations (hypothesis 4), i.e., “islands” of isolated demes, affects the probability of reciprocal monophyly of two samples and the chance that a gene tree matches the species tree [42], and explained the persistence of ancestral polymorphisms and the extreme divergence in *Achatinella* [43], *Systrophia* [44], and *Xerocrassa* [45]. In addition, in the case of Hygrophila, some studies found high divergence rates in *Physella* [46], and *Radix* [47], although no clear hypothesis has been proposed to explain this pattern.

### Analyses of selective pressures

Codon substitution models have been widely used to detect adaptive signatures affecting protein evolution [48]. First, we tested for the presence of positive selected codons across the alignments. All comparisons (M2a-M1a, M8-M7, M8-M8a) consistently favored positive selection models M2a and M8 in *cox1*, *cox2*, *cox3*, and *cob* (*p* < 0.05) (Table 2). However, the proportion of sites with ω > 1 was extremely low (Additional files 3 and 4). High ω-values in the positively selected genes can be explained by the presence of few synonymous sites affecting dN estimations. On the contrary, a context of strong negative selection could explain ω < 1 for most of the genes [49, 50].

**Table 2:** Site test of positive selection

	M1a (np = 3)	M2a (np = 5)	M7 (np = 3)	M8 (np = 5)	M8a (np = 4)	M2a/M1a (df = 2)	M8/M7 (df = 2)	M8/M8a (df = 1)
ATP6	−23368.8643	−23368.7771	−22291.9994	−22292.0038	−22292.0038	0.1744	−0.008956	0.0000
COX1	−30899.0024	−30854.9357	−29758.9356	−29704.0913	−29746.9794	**88.1334**	**109.6887**	**85.7763**
COX2	−18042.2493	−18015.6742	−17256.1223	−17221.3147	−17245.3928	**53.1501**	**69.6151**	**48.1561**
COX3	−21196.1015	−21158.9525	−20023.0527	−20010.8195	−20023.0034	**74.2980**	**24.4664**	**24.3678**
CYTB	−30416.7613	−30401.6179	−28984.2418	−28973.9145	−28984.1611	**30.2867**	**20.6547**	**20.4933**
ND1	−28047.2931	−28045.7290	−26771.4013	−26771.4065	−26771.4065	3.1282	−0.010268	0.0000
ND2	−36553.7114	−36553.7117	−35518.2839	−35518.2961	−35518.2961	−0.0006	−0.024316	0.0000
ND4	−47287.5457	−47287.5457	−45097.7072	−45097.4722	−45097.4722	0.0000	0.46994	0.0000
ND5	−58590.6941	−58589.0944	−55650.7195	−55650.7278	−55650.7278	3.1994	−0.016656	0.0000

The comparisons within the site models were M8 vs. M8a/M7, and M2a vs. M1a. Values in bold represent highly significant differences (*p* < 0.01) from the null model, np: number of parameters

*df* degrees of freedom

Positive selection tests based on either sites or branches only, are conservative for many genes [51]. This is because the test is only significant if the average ω > 1 holds true for all sites or all branches. However, one might expect that positive selection affects only specific sites in specific branches or lineages [52]. For these reasons, we used the branch-site test of positive selection [51] focusing on the branches leading to terrestrial taxa (foreground) within Panpulmonata. The alternative model (model A) fitted significantly better than the null model (model A1) in the genes *cox1*, *cox2*, *cob* and *nad5* (Additional file 5). From these four genes, only *cob* and *nad5* presented an ω-ratio higher than one and positively selected codons in the foreground (two sites in *cob* and six in *nad5*) (Table 3).

**Table 3:** Branch-site test of positive selection on the mitochondrial genes

Gene	Proportion of site classes under model A				dN / dS (ω) in the foreground (terrestrial taxa)				Positively selected sites
	0	1	2a	2b	0	1	2a	2b	BEB (pp > 0.95)
*cox1*	88.37	9.98	1.49	0.17	0.0073	1.0000	1.0000	1.0000	-
*cox2*	79.08	14.54	5.39	0.99	0.0278	1.0000	1.0000	1.0000	188
*cob*	85.13	11.90	2.60	0.36	0.0243	1.0000	**2.4988**	**2.4989**	006, 151
*nad5*	71.79	21.13	5.48	1.61	0.0559	1.0000	**2.1521**	**2.1521**	169, 308, 474, 478, 479, 512

Only the genes that showed a significant difference from the null model are shown. Values in bold represent a ω > 1

*BEB* Bayes Empirical Bayes algorithm, *pp* posterior probability

The branch-site model has been shown to detect ancient episodes of positive selection [53]. A potential problem of the test can be the saturation over long evolutionary times; however, simulations have shown that extreme sequence divergence does not generate false positives although it can lead to a high rate of false negatives, especially in older nodes [54]. In the same study, significant levels of positive selection (ω = 6 or 12) were detected at the radiation of bony vertebrates (Euteleostomi), approximately 400–500 Ma [55]. In our data, divergence times are lower than in the vertebrate study: Euthyneura and Panpulmonata probably diverged from their sister groups 250–350 Ma and 150–250 Ma ago, respectively; while panpulmonate clades with terrestrial taxa diverged more recently (Ellobioidea: 140–160 Ma; Stylommatophora: 100–150 Ma) [3, 5].

### Convergent adaptations related to realm shifts

The evolution of the lung in early panpulmonates, probably originating from the pallial cavity of an intertidal gilled-ancestor, was the key evolutionary innovation that allowed the diversification to non-marine realms [24]. Both Ellobioidea and Stylommatophora possess lungs, although they colonized the land in different times. Land invasions in Ellobioidea occurred at least twice, one within the genus *Pythia* (15–25 Ma) and the other in the subfamily Carychiinae (50–100 Ma) [8]; while terrestrialization in Stylommatophora appeared to be older (100–150 Ma) [5].

**Fig. 2:**
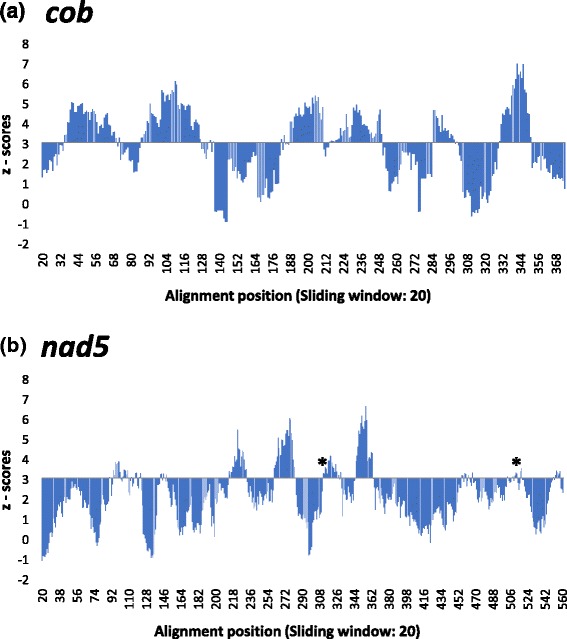
Detection of significant physicochemical amino acids changes using TreeSAAP. This analysis was performed on the genes that were shown to be under positive selection by PAML. Regions above the z-score of 3.09 were significantly different than assumed under neutrality. Only the highest significant category (8) is shown. This category represents a significant change in the equilibrium constant (ionization of COOH) property. **a** *cob*, **b** *nad5*. Asterisks indicate regions under positive selection according to PAML and TreeSAAP (sites 308 and 512)

Different genes appeared to be under positive selection (ω > 1) in terrestrial panpulmonate branches. While *cob* and *nad5* are both part of the OXPHOS pathway, they belong to different complexes (complex III and I, respectively), suggesting that adaptations occurred in several molecular targets. The observed non-synonymous mutations produced similar changes in the amino acid properties, albeit in different regions of each gene. Results from TreeSAAP for both *cob* and *nad5* showed that these changes alter the equilibrium constant (ionization of COOH) property (Fig. 2). This property may influence the protein efficiency reducing ROS production while increasing individual longevity [56]. Alterations in the equilibrium constant may have allowed organisms to better cope with abiotic stress conditions in the new hot, cold or dry habitats. For example, desiccation tolerance has been shown as a limiting factor for the invasion of dry habitats in thiarid freshwater snails [57]. Moreover, the activation of the antioxidant metabolism reducing ROS excess has been linked to desiccation tolerance in the algae *Mastocarpus stellatus* and *Porphyra columbina* occurring in the upper intertidal zone [58]. Since desiccation stress (or abiotic stress in general) is linked to metabolic activity and ROS production [59], this directly affects the invasion success of an evolutionary lineage.

The increase in metabolic efficiency has been also related with the terrestrial invasion in other animals, e. g., tetrapods, during the Devonian. Amphibians, lungfishes, and coelacanths presented significant changes in the same equilibrium constant (ionization of COOH) property suggesting an adaptation to increased oxygen levels and changing metabolic requirements [13]. These mutations affected both *cob* and *nad5* tetrapod genes. Also, it is noteworthy to mention that in the terrestrial panpulmonate *nad5* gene, the different approaches used by PAML and TreeSAAP found signatures of positive selection along with amino acid property changes in similar regions (Sites 308 and 512; Fig. 2, Table 3).

Changes at the molecular level could have also occurred in different taxa, so we compared the sites under positive selection in our data against previous studies to find sites with similar adaptive patterns (Tables 4 and 5). For *cob*, site 151 is located in an intermembrane domain. This site is homologous to site 158 in a previous cetacean-artiodactyl alignment (Table 4) and was revealed to be under selective pressure in cetaceans, also influencing the equilibrium constant of the cd2 intermembrane helix [20]. The authors proposed that non-synonymous changes in *cob* are related to increasing metabolic demands during cladogenesis. Similarly, adaptive evolution in *cob* has been related to the increase of energy metabolism in response to the evolution of flight in bats [60].

**Table 4:** Alignment section of the *cob* gene from Garvin et al. [17]

	Position in the alignment from Garvin et al. [17]					
	156	157	158	159	160	161
*Anguilla*	V	G	D	T	L	V
Argentenoidei	V	G	E	A	L	V
Baleanopteridae	I	G	N	T	L	V
Caprinae	I	G	T	N	L	V
Delphinidae	I	G	T	T	L	V
*Hypsiglena*	L	G	T	S	L	T
*Oryzias*	V	G	N	A	L	V
Otariidae	I	G	A	N	L	V
Phocidae	I	G	T	D	L	V
Primates	I	G	T	D	L	V
Salmonidae	V	G	N	A	L	V
*Takifugu*	V	G	N	T	L	V
Ursidae	I	G	T	D	L	V
*Bos taurus*	I	G	T	N	L	V
McClellan et al. [20]			158			
da Fonseca et al. [14]			158			
This work			151			

The corresponding homologous position in *Bos taurus* is shown. Homologous positions found under positive selection in other studies are shown below the alignment

**Table 5:** Alignment section of the *nad5* gene from Garvin et al. [17]

	Position in the alignment from Garvin et al. [17]					
	540	541	542	543	544	545
*Anguilla*	T	H	N	F	S	N
Argentenoidei	L	H	N	F	S	N
Baleanopteridae	**F**	S	K	F	S	T
Caprinae	T	F	K	F	S	N
Delphinidae	S	T	K	F	S	T
*Hypsiglena*	L	N	L	F	F	N
*Oryzias*	**T**	H	H	F	S	N
Otariidae	L	F	K	F	S	N
Phocidae	L	F	K	F	S	S
Hominidae	T	F	R	F	S	N
Salmonidae	T	H	N	F	S	N
*Takifugu*	P	H	H	F	S	N
Ursidae	P	F	K	F	S	N
*Escherichia coli*	NuoL527	NuoL528	NuoL529	NuoL530	NuoL531	NuoL532
Garvin et al. [19]	526					
Tomasco and Lessa [49]	519	520				
This work	474				478	479

Positions under positive selection according to Garvin et al. [17] are highlighted in bold. The corresponding homologous position in *Escherichia coli* is shown. Homologous positions found under positive selection in other studies are shown below the alignment

In case of *nad5*, site 474 is homologous to site 540 from the alignment of Garvin et al. [17] (Table 5). This site is positively selected in rorquals (Balaenopteridae) and salmons (Salmonidae). Site 474 is part of the biomechanical apparatus that generates the electrochemical gradient and it has been shown to be under positive selection in the Pacific salmon species [19] and in eutherians [14]. Also, site 474 corresponds to site 519 in subterranean rodents [49]. This codon position is positively selected only in lineages that independently colonized the subterranean niche, a habitat suggested to be energetically demanding [61]. Mutations in the NADH complex, especially in the transmembrane domains, may affect the proton pump activity of this complex [14]. These changes could facilitate the proton flow and improve the efficiency of ATP production - characteristics associated to increased energetic requirements in non-marine habitats [62].

## Conclusions

We represent evidence of positive selection on several amino acid positions in the mitochondrial complexes I (*nad5*) and III (*cob*). These episodes of positive selection occurred in independent branches of panpulmonates with terrestrial taxa (Ellobioidea and Stylommatophora), indicating their possible role during the invasion of the land realm. Most of these sites have been shown to be also under positive selection in several other taxa. Moreover, the general pattern suggests that non-synonymous mutations in both genes are probably linked to increased or altered metabolic requirements. An increased demand for energy due to the colonization of land and the necessity to cope with different abiotic stress conditions may have changed the physiological constraints in the early terrestrial panpulmonates and triggered functional adaptations at the mitochondrial level. Future studies can take into account the predicted codons and the information on the physicochemical changes to test whether these mutations also affect protein structure and function. New genomic information from panpulmonates will most likely reveal even more genes involved in metabolic and structural processes that were key to the colonization of the terrestrial realm.

## Methods

### Mitogenome sequencing

Our dataset comprises 50 complete mitochondrial genomes, and is representative of all described lineages within Euthyneura, except Acochlidia where no mitogenomes have been sequenced so far. We did not consider identical mitogenomes and mitogenomes that were not verified for biological accuracy by GenBank. Accession numbers for each sample are provided in the Additional file 6.

In addition, we used DNA previously isolated from specimens of *Arion rufus* (Linnaeus, 1758) [63], *Carychium tridentatum* (Risso, 1826) [64], and *Helicella itala* (Linnaeus, 1758) [65] for DNA shotgun sequencing. We followed the protocol described by Feldmeyer et al. [66] with some variations: 500 ng DNA per sample was used for library preparation, following the Roche GS FLX Titanium General Library Preparation specifications. Each sample was sequenced on 1/8 of a titanium plate on a 454 sequencer. It should be noted, that approximately 100 specimens of the microgastropod *C. tridentatum* originating from a single locality had to be pooled to obtain 500 ng of DNA.

### Mitogenome assembly and annotation

Newbler v2.0.1 (Roche) was used for contig assembly, with standard settings. Then, we subjected contigs to BlastN and BlastX searches against the mitochondrial genomes of closely related taxa. In addition, to close the remaining gaps after the assembly, we designed flanking primers using Geneious R7 [67]. Primer sequences can be found in Additional file 7. Sequence amplification was conducted using the following PCR conditions: Each 25 μL PCR mix included 1 μL (10 pmol) of each primer, 2.5 μL 10x PCR buffer, 2 μL (100 mM) MgCl2, 0.2 μL (20 mM) dNTPs, 0.3 μL Taq-polymerase (Fermentas), 1.5 μL (10 mg/mL) bovine serum albumin, 12.50 μL ddH2O and 4 μL template DNA in variable concentrations. Temperature conditions: 1 min at 95 °C, followed by 30 cycles of 30 s at 95 °C, 30 s at 52 °C and 30 s at 72 °C, and finally, 3 min at 72 °C. Visualization of PCR products was performed on a 1.4 % agarose gel. Amplicons were cleaned using the QIAquick PCR Purification Kit or the QIAquick Gel Extraction kit (Qiagen) whenever multiple bands were detected. Sanger sequencing was performed using the PCR primer pair (5 pmol) and the BigDye® Terminator v.3.1 Cycle Sequencing Kit (LifeTechnologies, Inc.) on an ABI 3730 capillary sequencer, using the facilities of the Senckenberg BiK-F Laboratory Centre, Frankfurt am Main.

Both, shotgun contigs and Sanger sequences were aligned in Geneious R7 to obtain the complete mitochondrial genomes. The complete mitogenome assemblies were annotated using the MITOS webserver [68]. This program also identified rRNA and tRNA genes. Additionally, we compared the results from MITOS to other annotation strategies like NCBI ORF Finder or Geneious R7, with similar results. Finally, we compared the new gene annotations against other panpulmonate mitochondrial genes to evaluate the length of the reading frames. Newly determined genomes were deposited into GenBank (accession numbers: KT626607, KT696545, KT696546).

### Sequence alignments

The 13 protein-coding genes (PCG) were translated into amino acids in Geneious R7 using the invertebrate mitochondrial genetic code, and then aligned using the MAFFT [69]. Ambiguous aligned regions were removed using Gblocks [70]. For downstream analyses, we did not use alignments that had a length below 150 aa (~450 nt) after Gblocks trimming. Thus, nine genes were selected: *atp6*, *cox1*, *cox2*, *cox3*, *cob*, *nad1*, *nad2*, *nad4*, *nad5*. Then, nucleotide sequences of these genes were aligned using TranslatorX [71]. This software aligns the nucleotides by codons taking into account the information from the amino acid alignment. Gblocks was used again in the codon-based alignment with less stringent parameters to trim flanking regions and long gaps. The concatenated alignment length is 9711 nt.

### Phylogenetic reconstructions

It has been shown that outgroup selection is important to conceal current hypothesis of euthyneuran phylogeny [21, 72]. Thus, we decided to follow Wägele et al. [28] choosing the “Lower Heterobranchia” clade (*Hydatina*, *Micromelo* and *Pupa*) as the outgroup. For tree reconstruction, we used only the first and second positions of the alignment in order to reduce saturation levels. The alignment length after removing the third position was 6474 nt. Data were partitioned by gene using the partition scheme suggested in PartitionFinder [73]. Maximum likelihood analyses were conducted in RAxML-HPC2 (8.0.9) [74, 75] implemented on XSEDE [76] (CIPRES Science Gateway). We followed the “hard and slow way” suggestions indicated in the manual and selected the best-likelihood tree after 1000 independent runs. Then, branch support was evaluated using bootstrapping with 1000 replicates, and confidence values were drawn in the best-scoring tree. Bayesian inference was conducted in MrBayes v3.2.2 [77] on XSEDE (CIPRES). Two simultaneous Monte Carlo Markov Chains (MCMC) were run, with the following parameters: eight chains of 50 million generations each, sampling every 1000 generations and a burn-in of 25 %. Tracer 1.6 [78] was used to evaluate effective sample sizes (ESS > 200). We assume that a bootstrap value of >70 % [79] and a posterior probability of > 0.95 [80] are evidence of significant nodal support.

### Patterns of evolutionary rates

Relative evolutionary rates were calculated in the software MEGA6 [81], using the nucleotide and amino acid information following the procedure described by Merker et al. [82]. The rates are scaled such that the average evolutionary rate across all sites is 1. Sites with a rate lower than 1 evolve slower than the average while sites with a rate higher than 1 evolve faster than the average. The relative rates were estimated under the General Time Reversible (GTR) model (+Γ) for nucleotide sequences (complete concatenated alignment: 9711 nt.) and under the mtREV model (+Γ) for amino acid sequences. The relative rates were scaled in windows with a size of 30 for nucleotides and 10 for amino acids, using the R package zoo [83]. Finally, we compared the amino acid divergence relative to the nucleotide divergence among main clades. Pairwise nucleotide and amino acid distances were calculated under the previously described substitution models.

### Analysis of selective pressures

The CODEML program from PAML v4.8a [84] was used to analyze positive selection in each mitochondrial gene. PAML estimates the omega ratio (ω = dN/dS) using maximum likelihood. The omega ratio compares non-synonymous (dN) against synonymous (dS) substitutions per site. Assuming neutrality, ω -values are equal to one; however, ω > 1 is expected if the gene undergoes adapting molecular evolution. In the latter scenario, non-synonymous mutations offer fitness advantages to the individual and have higher fixation probabilities than synonymous mutations [85].

The maximum likelihood topology was set as the guide tree. We re-estimated branch lengths on the tree using codon model M0 (one-ratio) and used them as fixed when fitting the site and branch-site models. In the site models, the ω-ratio is allowed to vary among codons in the alignment [85], while in the branch-site models, the test focuses on the so-called foreground branches [50]. Specifically, we tested branches leading to the air-breathing land snails and slugs (Stylommatophora) and to the terrestrial *Carychium* (Ellobioidea).

We evaluated site models using a likelihood-ratio test (LRT). First, we compared the selection model (M2a; model = 0, NSites = 2, fix_omega = 0, omega = 5) against the nearly neutral model (M1a; model = 0, NSites = 1, fix_omega = 0, omega = 1) to detect signatures of ω > 1. Then, we used models that calculate ω from a beta distribution. Model M8 (model = 0, NSites = 8, fix_omega = 0, omega = 5, 10 equal class proportions plus one class with ω > 1) was compared against either model M7 (model = 0, NSites = 7, fix_omega = 0, omega = 1, 10 equal class proportions) or model M8a (model = 0, NSites = 8, fix_omega = 0, omega = 1, 10 equal class proportions plus one class with ω = 1).

For the branch-site test we compared the model A (model = 2, NSsites = 2, fix_omega = 0, omega = 5) against the null model A (model = 2, NSsites = 2, fix_omega = 1, omega = 1). The LRT was calculated as follows, 2*(lnL H1 – lnL H0). The Bayes Empirical Bayes (BEB) algorithm implemented in CODEML was used to calculate posterior probabilities of positive selected sites.

Genes detected to be positively selected in the branch-site test were then analyzed in TreeSAAP [86]. This software identifies significant physicochemical changes comparing the distribution of observed changes inferred from a phylogenetic tree against the random distribution of changes under neutrality. The magnitude of the change is rated from 1 (most conservative) to 8 (most radical). A highly significant z-score calculated in TreeSAAP (z > 3.09, *p* < 0.01) indicates more non-synonymous substitutions than assumed under the neutral model [87]. We followed the suggestions from George and Blieck [13] to increase the accuracy of the test and lower the rate of false positives. Thus, we considered only the most radical changes (category 8, *p* < 0.01) as significant. Moreover, we focused only on 20 of the 31 amino acid properties available in the software.

### Alignment comparisons with previous studies

Almost all previous work on mitochondrial molecular adaptation has been done on vertebrates. We used the amino acid alignments from a recent review by Garvin et al. [17] to evaluate whether the positive selected sites found in our study are also present in a broader taxonomic context and could present a biological function. Mitochondrial sequences of *cob* and *nad5* reported by Garvin et al. [17] and references therein [14, 19, 20, 49] were downloaded and aligned using the global homology algorithm g-insi in MAFFT [69].

## Additional files

Additional file 1: Evolutionary rates for nucleotides (NT; blue line) and amino acids (AA; red line) in the euthyneuran mitogenomes. Rates are scaled such that the average evolutionary rate across all sites is 1 (red line). The x-axis shows amino acid positions in the final concatenated alignment. (PDF 481 kb) Additional file 2: Amino acid divergence versus nucleotide divergence in mitochondrial genomes of euthyneuran gastropods. Clades are differentiated by colors and symbols as shown in the legend. (PDF 205 kb) Additional file 3: Comparison of models M0 (one-ratio), M1a (nearly neutral), and M2a (positive selection), and. Values in bold represent highly significant differences (*p* < 0.01) from the null model. LRT: Likelihood ratio test, df: degrees of freedom, lnL: log likelihood, np: number of parameters. (XLSX 14 kb) Additional file 4: Comparison of models M7, M8 and M8a. Values in bold represent highly significant differences (*p* < 0.01) from the null model. LRT: Likelihood ratio test, df: degrees of freedom, lnL: log likelihood, np: number of parameters. (XLSX 14 kb) Additional file 5: Branch-site test of positive selection on the mitochondrial genes (Model A vs. Null model). Values in bold represent highly significant differences (*p* < 0.01) from the null model. LRT: Likelihood ratio test, df: degrees of freedom, lnL: log likelihood, np: number of parameters. (XLSX 10 kb) Additional file 6: GenBank accession numbers of the 50 sequences used in this study. (XLSX 12 kb) Additional file 7: Primer sequences used to close the gaps and complete the mitochondrial genomes of *Arion* and *Carychium*. (XLSX 9 kb)
