# Supplementary figures and images for: Positive selection on panpulmonate mitogenomes provide new clues on adaptations to terrestrial life
Source: BMC Evol Biol. 2016 Aug 22;16(1):164. doi: 10.1186/s12862-016-0735-8 (PMC4994307; doi:10.1186/s12862-016-0735-8)

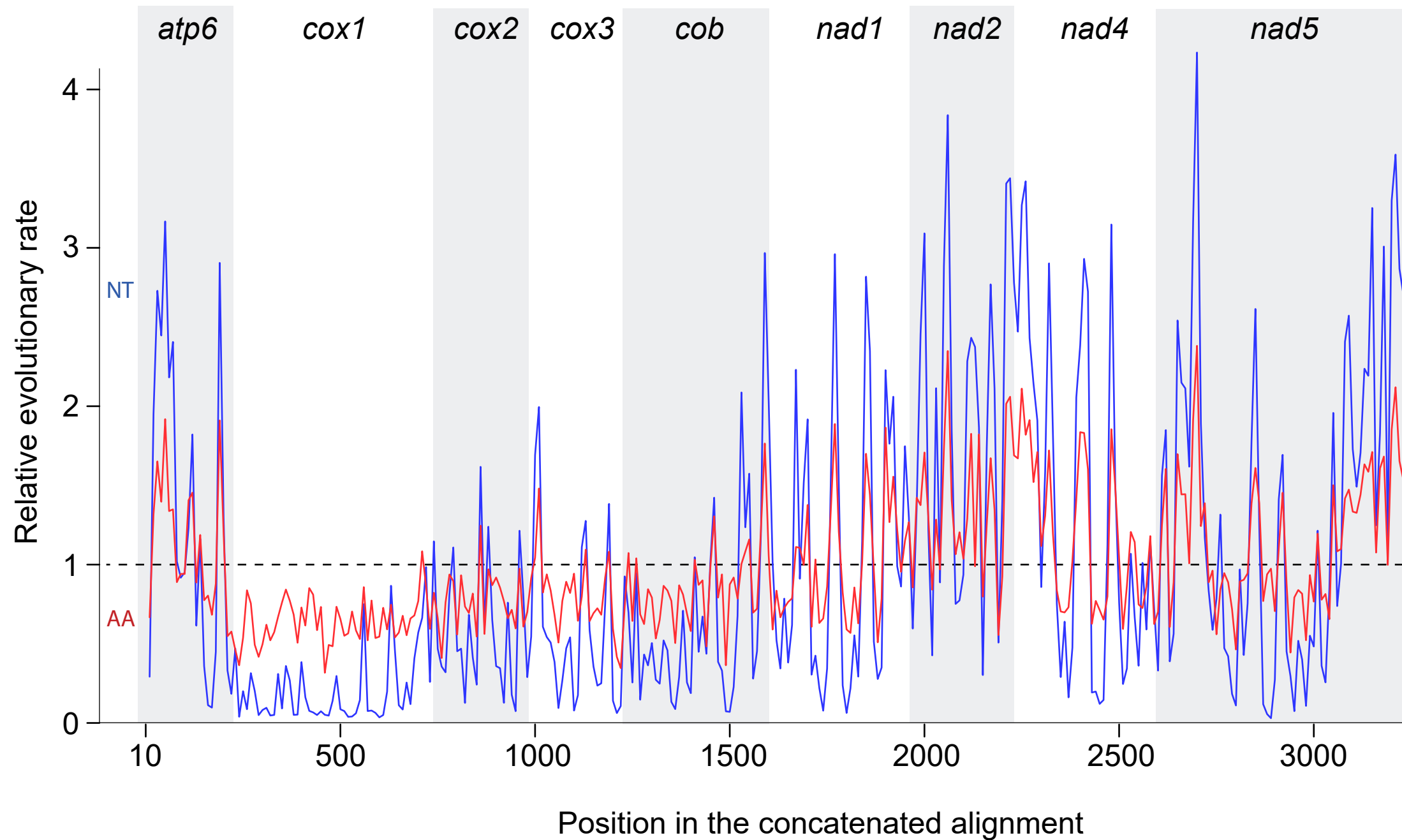

Supplement: Additional file 1: — Evolutionary rates for nucleotides (NT; blue line) and amino acids (AA; red line) in the euthyneuran mitogenomes. Rates are scaled such that the average evolutionary rate across all sites is 1 (red line). The x-axis shows amino acid positions in the final concatenated alignment. (PDF 481 kb) [file 12862_2016_735_MOESM1_ESM.pdf]

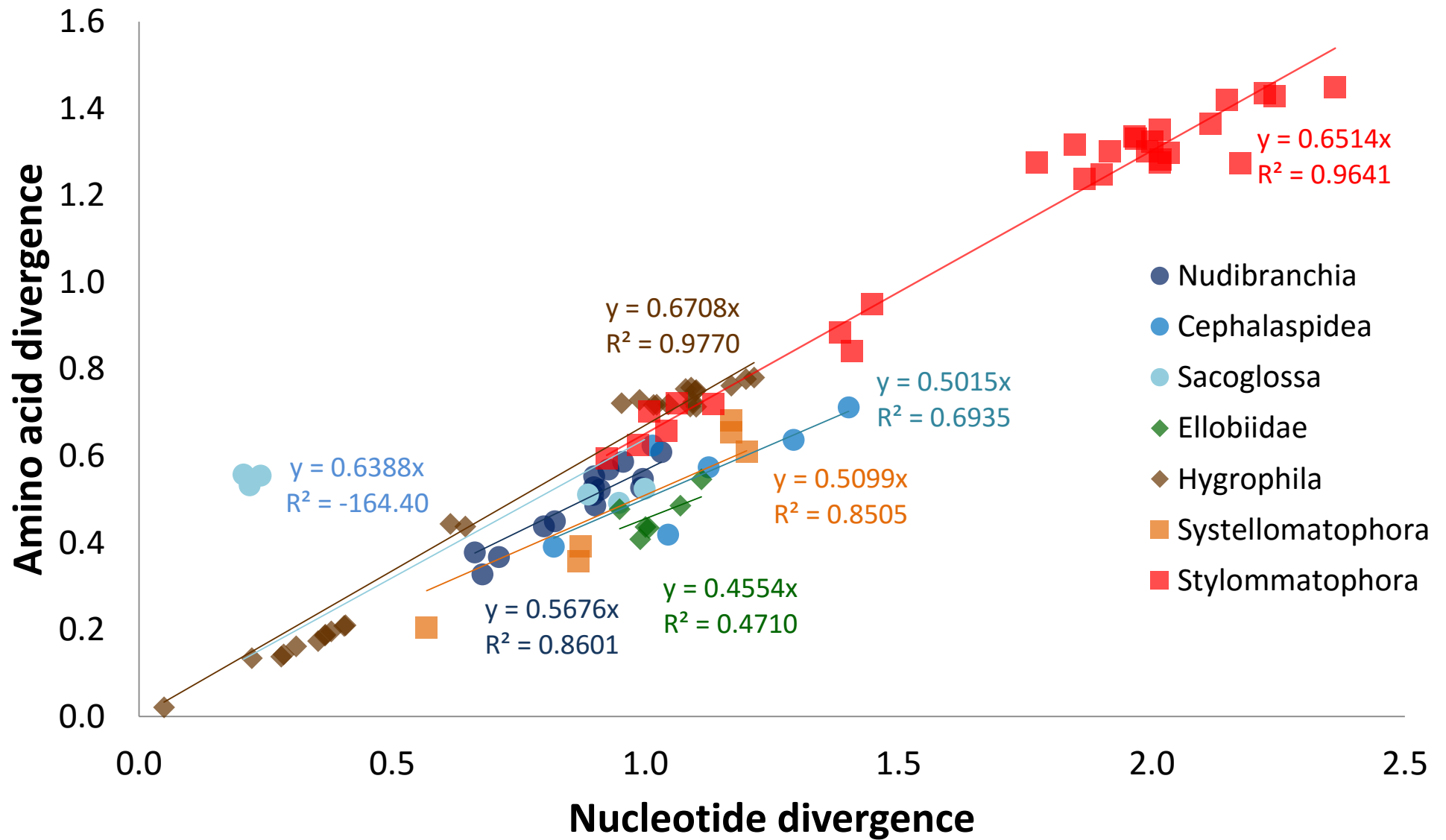

Supplement: Additional file 2: — Amino acid divergence versus nucleotide divergence in mitochondrial genomes of euthyneuran gastropods. Clades are differentiated by colors and symbols as shown in the legend. (PDF 205 kb) [file 12862_2016_735_MOESM2_ESM.pdf]
